# Delay in antibiotic therapy results in fatal disease outcome in murine pneumococcal pneumonia

**DOI:** 10.1186/s13054-018-2224-5

**Published:** 2018-11-01

**Authors:** Sarah Berger, Cengiz Goekeri, Shishir K. Gupta, Julio Vera, Kristina Dietert, Ulrike Behrendt, Jasmin Lienau, Sandra-Maria Wienhold, Achim D. Gruber, Norbert Suttorp, Martin Witzenrath, Geraldine Nouailles

**Affiliations:** 1 Division of Pulmonary Inflammation, Charité — Universitätsmedizin Berlin, corporate member of Freie Universität Berlin, Humboldt-Universität zu Berlin, and Berlin Institute of Health, Charitéplatz 1, 10117 Berlin, Germany; 2 Department of Dermatology, Laboratory of Systems Tumor Immunology, Friedrich-Alexander-Universität Erlangen-Nürnberg, Erlangen, Germany; 3 Department of Veterinary Pathology, Freie Universität Berlin, Berlin, Germany; 4 Department of Infectious Diseases and Respiratory Medicine, Charité — Universitätsmedizin Berlin, corporate member of Freie Universität Berlin, Humboldt-Universität zu Berlin, and Berlin Institute of Health, Berlin, Germany

**Keywords:** *Streptococcus pneumoniae*, Blood–air barrier, Bacterial pneumonia, Acute lung injury, Ampicillin, Innate immunity

## Abstract

**Background:**

Community-acquired pneumonia (CAP) remains a major cause of death worldwide. Mechanisms underlying the detrimental outcome despite adequate antibiotic therapy and comorbidity management are still not fully understood.

**Methods:**

To model timely versus delayed antibiotic therapy in patients, mice with pneumococcal pneumonia received ampicillin twice a day starting early (24 h) or late (48 h) after infection. Clinical readouts and local and systemic inflammatory mediators after early and late antibiotic intervention were examined.

**Results:**

Early antibiotic intervention rescued mice, limited clinical symptoms and restored fitness, whereas delayed therapy resulted in high mortality rates. Recruitment of innate immune cells remained unaffected by antibiotic therapy. However, both early and late antibiotic intervention dampened local levels of inflammatory mediators in the alveolar spaces. Early treatment protected from barrier breakdown, and reduced levels of vascular endothelial growth factor (VEGF) and perivascular and alveolar edema formation. In contrast, at 48 h post infection, increased pulmonary leakage was apparent and not reversed by late antibiotic treatment. Concurrently, levels of VEGF remained high and no beneficial effect on edema formation was evident despite therapy. Moreover, early but not late treatment protected mice from a vast systemic inflammatory response.

**Conclusions:**

Our data show that only early antibiotic therapy, administered prior to breakdown of the alveolar–capillary barrier and systemic inflammation, led to restored fitness and rescued mice from fatal streptococcal pneumonia. The findings highlight the importance of identifying CAP patients prior to lung barrier failure and systemic inflammation and of handling CAP as a medical emergency.

**Electronic supplementary material:**

The online version of this article (10.1186/s13054-018-2224-5) contains supplementary material, which is available to authorized users.

## Background

Community-acquired pneumonia (CAP) is a significant cause of morbidity and mortality worldwide, with *Streptococcus pneumoniae* being the most prevalent causative pathogen [1, 2]. Since the 1950s, the in-hospital mortality rate of CAP has remained about 12–13% in most high-income countries [3]. Severe forms of CAP necessitate admission to the intensive care unit (ICU) and result in mortality rates ranging from 18 to 38% [4–6]. Antibiotic intervention within 4 h of hospital arrival is associated with reduced mortality compared to a delayed start of treatment in CAP [7, 8]. To improve survival in severe pneumonia, CAP is nowadays described as a medical emergency and early and aggressive treatment is therefore proposed on an empiric basis [9–14]. However, the pathophysiological differences in the course of pneumonia resulting from early as opposed to late treatment are unknown.

Differences in survival of CAP patients receiving antibiotic therapy result from a wide range of contributors, which can be pathogen, drug or host related. The host’s immune response may aggravate detrimental pulmonary barrier failure and lung edema development [15–19]. Particularly, activation of lung resident cells (e.g., alveolar macrophages and epithelial cells) by pathogen-associated molecular patterns (PAMPs) results in local inflammation, which in turn promotes attraction of inflammatory cells like polymorphonuclear leukocytes (PMNs) into the lungs [20–22]. As professional phagocytes, PMNs are crucial for antimicrobial defense; however, PMNs also cause host tissue injury, leading to increased permeability of the alveolar–capillary barrier [23, 24]. As a further consequence of pulmonary barrier failure, CAP can progress to life-threatening sepsis and multiorgan dysfunction [25]. However, specific reasons for host-related differences in survival that depend on timely versus delayed antibiotic treatment remain unclear to date. Analysis of processes contributing to host-related therapy failure and high mortality rates due to delayed treatment are needed to foster the development of new adjunctive therapies. We hypothesized that antibiotic treatment decelerates exaggerated immune responses, but does not relevantly reduce established lung barrier dysfunction and lung edema. In the current study, we performed in-depth examination of mice with severe pneumococcal pneumonia receiving early versus late antibiotic treatment.

## Methods

A detailed methodology and materials are presented in Additional file 1: Materials and methods.

### Study approval

Female C57BL/6 N mice (8–10 weeks old; Charles River, Germany) were housed under specific-pathogen-free conditions. All animal studies have been ethically reviewed and approved by the State Office for Health and Social Services, Berlin, Germany.

### Murine bacterial pneumonia

Mice were transnasally inoculated as previously described in detail [26] with 5 × 10^6^ colony-forming units (CFUs) of *S. pneumoniae* serotype 3 in 20 μl PBS. Control mice received sham infection (PBS). Starting at 24 h or 48 h p.i., ampicillin (0.4 mg/mouse) or 0.9% NaCl (solvent control) was injected intraperitoneally every 12 h (Additional file 1: Figure S1). Body weight, temperature and murine pneumonia symptoms (scaling from 0 (absent) to 1 (present) and 2 (severe)) were assessed twice daily. Detailed information about the scoring system for murine pneumonia symptoms is presented in Additional file 1: Materials and methods, and Table S3. The summary of scored symptoms provides individual clinical scores. Mice meeting the inclusion criteria at 24 h p.i. (body weight loss more than 10% and/or body temperature < 37.0 °C) were analyzed. Numbers of analyzed mice per group per time point are summarized in Additional file 1: Tables S1 and S2. At defined endpoints, mice were anesthetized and exsanguinated prior to analysis. EDTA-blood was used for hemogram (measured with Scil Vet abc; scil animal care company GmbH) and bacterial load determination. The remaining blood was collected in serum separator tubes (BD) and sera were stored at − 80 °C.

### Bronchoalveolar lavage

Mice were sacrificed prior to lavage. Airways were washed twice with 800 μl PBS. Bronchoalveolar lavage (BAL) suspensions were used to determine the bacterial burden. Supernatant (BAL fluid (BALF)) was frozen at − 80 °C. BAL cells were used for leukocyte differentiation.

### Bacterial loads

Serial dilutions of EDTA-blood and BAL suspensions were plated onto Columbia agar with 5% sheep blood (BD) and incubated for 24 h at 37 °C under 5% CO_2_ prior to CFU counting.

### Histological analysis

Mice were sacrificed prior to histological analysis. Lungs were immersion-fixed in 4% buffered formalin, embedded in paraffin, cut into 2-μm sections and stained with hematoxylin and eosin (H&E) as described previously [27, 28]. Lung inflammation was scored based on consideration of specific parameters graded on a scale of 0 (absent) to 4 (severe), including the degree of inflammation, infiltration of neutrophils, pleuritis and steatitis. A lung edema score was assessed as a sum of distribution and degree of interstitial (perivascular) and alveolar edema graded on a scale of 0 (absent) to 5 (massive).

### Blood clinical analytes

AST and urea levels were measured using Cobas-8000-C701 (Roche) by LABOKLIN (Bad Kissingen, Germany).

### Leukocyte differentiation

BAL cells were preincubated with blocking antibody and stained with anti-CD11c, anti-CD11b, anti-F4/80, anti-Ly6G, anti-Ly6C and anti-MHCII antibodies. Cells were measured and analyzed with FACS Canto II (BD) and FACSDiva software. Cell numbers were calculated using CountBright Absolute Counting Beads (ThermoFisher Scientific).

### Pulmonary vascular leakage

Mouse serum albumin (MSA) levels in BALF and serum were measured by ELISA (BETHYL). The alveolar–capillary barrier permeability index was calculated as the ratio of BALF/serum MSA.

### Multiplex assay and ELISA

Mouse cytokine/chemokine levels were measured in BALF and serum with the ProcartaPlex Custom Mix & Match according to the manufacturer’s instructions (AffymetrixBioscience). BALF levels of CXCL2 and CXCL5 were measured by ELISA (RD).

### Cytokine-immune cell network

Details for construction of the network are described in Additional file 1: Materials and methods.

### Data analyses

GraphPad Prism 6 software was used for statistical analyses. Survival curves were analyzed using the Kaplan–Meier method and log-rank (Mantel–Cox) test. The Kruskal–Wallis test/Dunn’s multiple comparisons test and one-way ANOVA/Dunnett’s multiple comparisons test were used for comparison to *S. pneumoniae*-infected mice at the start of therapy. Grouped analyses were performed using two-way ANOVA/Sidak’s multiple comparison test. *p* < 0.05 was considered significant.

## Results

### Late yet efficient antibiotic treatment did not rescue *S. pneumoniae*-infected mice

**Fig. 1:**
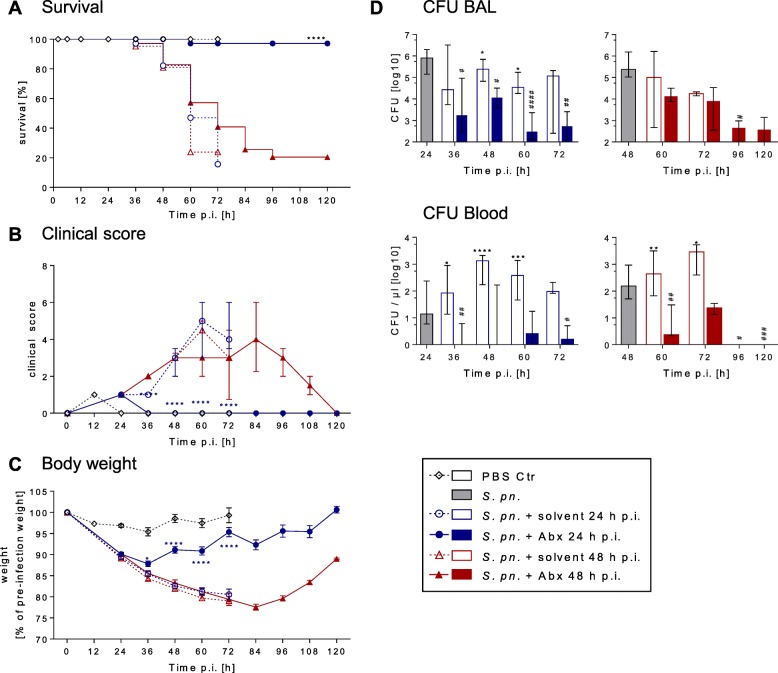
Late antibiotic therapy failed to rescue *S. pneumoniae*-infected mice. Mice were infected with *S. pneumoniae* and assigned equally to groups and analysis time points (*n*_total_ = 9 per time point)*.* Starting 24 h or 48 h p.i., intervention groups were treated with ampicillin. As controls, mice were sham infected (PBS; *n*_total_ = 7 per time point) or treated with solvent (0.9% NaCl). Survival, murine clinical score and weight assessed for all mice until designated analysis time point. Mice surviving until designated analysis time point were sacrificed for BAL and blood sampling (number analyzed per time point presented in Additional file 1: Table S1). **a** Kaplan–Meier curves showing survival of experimental groups, log-rank test. **b** Murine clinical disease score. Median and 25–75% interquartile range (IQR). **c** Relative weight curves. Mean ± SEM. **d** Bacterial burden in BAL and blood of respective mice analyzed at indicated time points. Median and 25–75% IQR. **a**–**d** Results pooled from three independent experiments per time point. **b**–**d** Two-way ANOVA/Sidak’s multiple comparisons test for comparison of ampicillin versus solvent treatment. Kruskal–Wallis test/Dunn’s multiple comparisons test for comparison to *S. pneumoniae*-infected mice at therapy start. *Significant difference between groups at time point, #significant difference from therapy start: *^/#^*p* < 0.05, **^/##^*p* < 0.01, ***^/###^*p* < 0.001 and ****^/####^*p* < 0.0001. Abx antibiotics, BAL bronchoalveolar lavage, CFU colony forming unit, Ctr control, PBS phosphate buffered saline, p.i. post infection, *S*. *pn*. *Streptococcus pneumoniae*

To improve understanding of clinical deterioration of individuals with bacterial pneumonia despite adequate antibiotic therapy, *S. pneumoniae*-infected mice were treated with ampicillin starting at different times p.i. Mice receiving ampicillin therapy starting 24 h p.i. showed a significantly higher survival rate (97.1%) than animals with late (48 h p.i.) therapy (20.4%) (Fig. 1a). Clinical signs of murine pneumonia like disheveled fur or an accelerated breathing rate and behavioral or somatic changes (Fig. 1b), weight loss (Fig. 1c), decrease in body temperature (Additional file 2: Figure S2), as well as lung infiltrates, assessed by histology (Additional file 2: Figure S2B), were already apparent at 24 h p.i. In line with superior survival rates, early antibiotic therapy led to normalization of body temperature and murine clinical scores within 12 h and increased body weight within 24 h. Mice receiving ampicillin from 48 h p.i. onward failed to restore their body weight and to reverse their murine clinical score within the following 48 h (Fig. 1b, c). Hence, for accurate interpretation of results at 72 h p.i. and beyond, the poor survival rates of untreated and late-treated groups (below 25%, Additional file 1: Tables S1 and S2) need consideration.

Notably, antibiotic therapy failure was not responsible for different outcomes in survival. At 24 h and 48 h p.i., before initiation of an antibiotic regimen, all mice analyzed at these time points had developed bacteremia. Within 12 h both regimens reduced the mean bacterial load in the blood by over 99% and in the alveolar spaces by over 85% (Fig. 1d, Additional file 2: Figure S3A).

### Antibiotic treatment had no major impact on innate leukocyte kinetics in BAL and blood

**Fig. 2:**
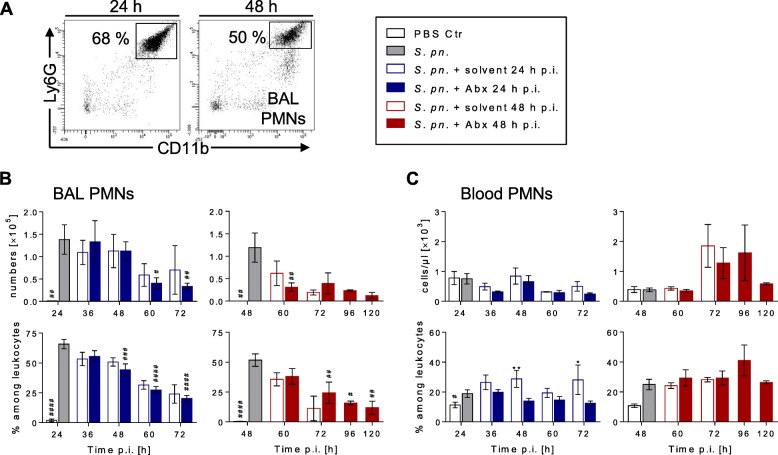
Antibiotic therapy has no impact on alveolar neutrophil recruitment. Mice were infected with *S. pneumoniae* and assigned equally to groups and analysis time points (*n*_total_ = 9 per time point)*.* Starting 24 h or 48 h p.i., intervention groups were treated with ampicillin. As controls, mice were sham infected (PBS; *n*_total_ = 7 per time point) or treated with solvent (0.9% NaCl). Mice surviving until designated analysis time point were sacrificed for BAL and blood sampling (number analyzed per time point presented in Additional file 1: Table S1). **a** Representative dot blots illustrating gating of innate BAL PMN populations at 24 h and 48 h p.i. **b** Numbers and frequencies of PMNs in BAL of mice analyzed at indicated time points p.i., quantified by flow cytometry. **c** Numbers and frequencies of PMNs measured in EDTA-blood by Scil Vet abc hematology analysis of respective mice. **b, c** Results pooled from three independent experiments per time point. Mean ± SEM. Two-way ANOVA/Sidak’s multiple comparisons test for comparison of ampicillin versus solvent treatment. One-way ANOVA/Dunnett’s multiple comparisons test for comparison to *S. pneumoniae*-infected mice at therapy start. *Significant difference between groups at time point, #significant difference from therapy start: *^/#^*p* < 0.05, **^/##^*p* < 0.01, ^###^*p* < 0.001 and ^####^*p* < 0.0001. Abx antibiotics, BAL bronchoalveolar lavage, Ctr control, PBS phosphate buffered saline, p.i. post infection, PMN polymorphonuclear leukocyte, *S*. *pn*. *Streptococcus pneumoniae*

Resident and recruited innate immune cells participate in defense against pulmonary pathogens [29] and may be influenced by antibiotic treatment. Consequently, we analyzed kinetics of PMNs, alveolar macrophages and Ly6C^hi^ inflammatory macrophages during infection and therapy (gating strategy; Additional file 2: Figure S4). After an initial recruitment wave, the number and frequency of BAL PMNs steadily declined from 24 h p.i. onward in treated and control groups (Fig. 2a, b, Additional file 2: Figure S3B). In the blood, however, frequencies but not total numbers of neutrophils (Fig. 2c, Additional file 2: Figure S3B) were significantly lower after early antibiotic intervention compared to the solvent-treated control group, whereas no difference was detected after late treatment. Likewise, antibiotic treatment did not affect the population size of alveolar macrophages, which remained unaffected throughout the infection course. Recruitment of Ly6C^hi^ inflammatory macrophages into alveolar spaces significantly increased following infection and remained unaltered by antibiotic therapy (Additional file 2: Figure S5A, B). Kinetics of blood monocyte number and frequency remained unaffected by antibiotic treatment. Similar results were obtained for eosinophils and platelets (Additional file 2: Figure S5C).

### Antibiotic treatment suppressed lung inflammation

**Fig. 3:**
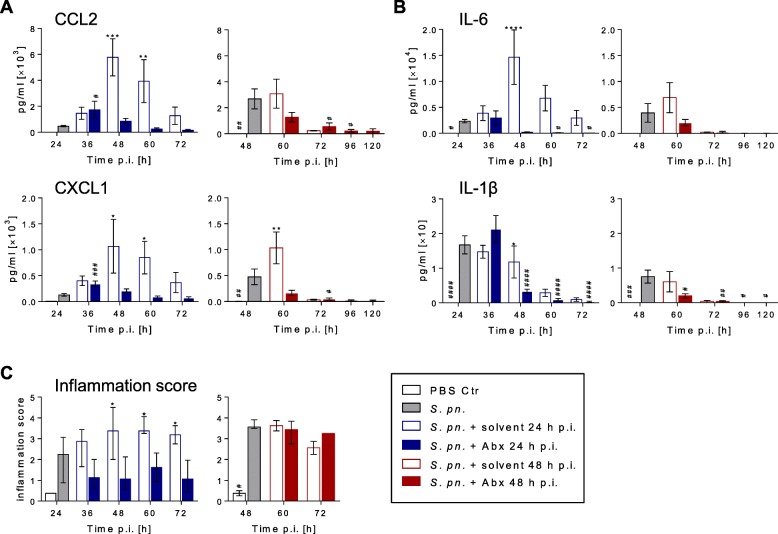
Antibiotic therapy reduced levels of alveolar inflammatory mediators. Mice were infected with *S. pneumoniae* and assigned equally to groups and analysis time points (**a**, **b** *n*_total_ = 9, **c** *n*_total_ = 4 per time point)*.* Starting 24 h or 48 h p.i., intervention groups were treated with ampicillin. As controls, mice were sham infected (PBS; **a**, **b** *n*_total_ = 7, **c** *n*_total_ = 4 per time point) or treated with solvent (0.9% NaCl). Mice surviving until designated analysis time point were sacrificed for BAL sampling (number analyzed per time point presented in Additional file 1: Table S1) or histopathological analysis (number analyzed per time point presented in Additional file 1: Table S2). **a** Chemokine and **b** cytokine protein levels in BAL fluid measured by multiplex analysis or ELISA. **a, b** Results pooled from three independent experiments per time point. Mean ± SEM. **c** Lung inflammation score calculated from specified histopathological parameters displaying distribution, severity and main character of lung lesions. Results pooled from two independent experiments per time point. Median and 25–75% interquartile range. **a–c** Two-way ANOVA/Sidak’s multiple comparisons test for comparison of ampicillin versus solvent treatment. **a**, **b** One-way ANOVA/Dunnett’s multiple comparisons test and **c** Kruskal–Wallis test/Dunn’s multiple comparisons test for comparison to *S. pneumoniae*-infected mice at therapy start. *Significant difference between groups at time point, #significant difference from therapy start: *^/#^ *p* < 0.05, **^/##^ *p* < 0.01, ***^/###^ *p* < 0.001 and ****^/####^ *p* < 0.0001. Abx antibiotics, Ctr control, IL interleukin, PBS phosphate buffered saline, p.i. post infection, *S*. *pn*. *Streptococcus pneumoniae*

Alveolar concentrations of chemokines and growth factors were measured in BALF (Fig. 3a, Additional file 2: Figures S3C and S6A). Levels of monocyte-recruiting chemokines CCL3 and CCL2 decreased within 24 h after antibiotic therapy compared to the concentration at treatment start. Notably, only after an early therapy start were significant differences between ampicillin-treated and solvent-treated mice with pneumonia observed for CCL2. Kinetics of neutrophil growth factor G-CSF levels resembled the kinetics of CCL2. After an initial increase in GM-CSF levels following an early therapy start, concentrations in BALF decreased with antibiotic therapy. CXCL1/KC, CXCL2/MIP-2 and CXCL5/LIX, homologs of human IL-8, are responsible for alveolar neutrophil recruitment [30]. Infected, solvent-treated mice showed an infection-time-dependent increase in CXCL1, CXCL2 and CXCL5. Notably, antibiotic intervention solely inhibited the increase of CXCL1 and CXCL2, but not CXCL5.

Both early and late antibiotic therapy led to a reduction in the local inflammatory cytokines IL-6, IL-1β, TNF-α and IFN-γ in the BALF. Likewise, the anti-inflammatory cytokine IL-10 decreased after early and late antibiotic therapy. On the contrary, IL-12p40 levels increased within 12 h after early therapy start and decreased after late antibiotic intervention (Fig. 3b, Additional file 2: Figures S3C and S6B). In line with these observations, the lung inflammation score, composed of specified histopathological inflammation parameters, was significantly decreased after early antibiotic therapy but remained unchanged after late antibiotic therapy (Fig. 3c, Additional file 2: Figures S3D and S7). Particularly, early but not late antibiotic intervention prevented development of pleuritis and steatitis of the adjacent mediastinal adipose tissue but did not show any impact on the innate cell recruitment into the lungs.

### Network analysis of alveolar immune cells and local effector molecules illustrated pronounced downregulation of cytokines by antibiotic treatment

The aim of the network analysis was to visualize and summarize consequences of early versus late antibiotic therapy on local cellular and cytokine/chemokine responses (Additional file 2: Figure S8). Hereby, we illustrate that recruitment of innate cells (Additional file 2: Figure S8, left) was independent of antibiotic treatment and treatment commencement. Cellular effector mechanisms such as cytokine secretion of IFN-γ, IL-1β, IL-6 and TNF-α were also strongly reduced by antibiotic treatment, while secretions of neutrophil-attracting chemokines (e.g., CXCL1, CXCL2, CXCL5 and G-CSF) were only modestly affected (Additional file 2: Figure S8, right).

### Early antibiotic treatment prevented systemic inflammatory responses

**Fig. 4:**
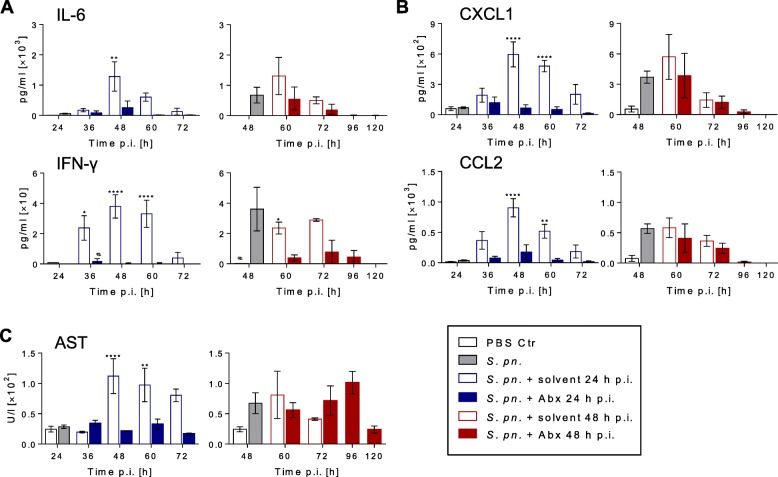
Early antibiotic treatment prevented systemic inflammation. Mice were infected with *S. pneumoniae* and assigned equally to groups and analysis time points (*n*_total_ = 9 per time point)*.* Starting 24 h or 48 h p.i., intervention groups were treated with ampicillin. As controls, mice were sham infected (PBS; *n*_total_ = 7 per time point) or treated with solvent (0.9% NaCl). Mice surviving until designated analysis time point were sacrificed for blood sampling (number analyzed per time point presented in Additional file 1: Table S1). **a** Cytokine and **b** chemokine protein levels in serum measured by multiplex analysis. **c** Serum AST levels measured by Cobas 8000 C701. **a–c** Results pooled from three independent experiments per time point. Mean ± SEM. Two-way ANOVA/Sidak’s multiple comparisons test for comparison of ampicillin versus solvent treatment. One-way ANOVA/Dunnett’s multiple comparisons test for comparison to *S. pneumoniae*-infected mice at therapy start. *Significant difference between groups at time point, #significant difference from therapy start: *^/#^*p* < 0.05, **p < 0.01 and ****p < 0.0001. Abx antibiotics, AST aspartate aminotransferase, Ctr control, IFN interferon, IL interleukin, PBS phosphate buffered saline, p.i. post infection, *S*. *pn*. *Streptococcus pneumoniae*

Following local pulmonary inflammation and bacteremia, the development of a systemic inflammatory response in untreated yet infected mice was observed. At 24 h p.i., prior to early antibiotic treatment, systemic cytokines were close to basal levels, although bacteremia was established. Notably, treatment with antibiotics starting 24 h p.i. prevented upsurge of inflammatory mediators such as IL-6, TNF-α and IFN-γ in serum compared to untreated yet infected mice. Late antibiotic therapy only led to a significant reduction in serum levels of IFN-γ, but basal levels were not reached. Systemic IL-10 concentrations were rarely detectable (Fig. 4a, Additional file 2: Figures S3E and S9A).

Generally, kinetics of systemic proinflammatory chemokines and growth factors highly resembled kinetics of systemic proinflammatory cytokines (Fig. 4a, b, Additional file 2: Figures S3E and S9A, B). Similar to systemic cytokine concentrations, systemic levels of chemokines and G-CSF increased later than in alveolar spaces.

At 24 h p.i., systemic chemokine levels were five times (CXCL1) to 90 times (CCL3) lower than prior late antibiotic therapy at 48 h p.i. Early antibiotic intervention prevented dramatic increases of G-CSF, CCL2 and CXCL1 in serum, with significant differences from the respective solvent-treated control groups. Early therapy did not lead to a significant change in CCL3 levels. After late antibiotic therapy, systemic CXCL1, G-CSF and CCL2 concentrations decreased, but no significant differences from the respective control groups were observed (Fig. 4b, Additional file 2: Figures S3E and 9B). Systemic levels of GM-CSF were below the detection limit (data not shown). To determine the functional integrity of the liver and kidneys, aspartate aminotransferase (AST) and urea were measured in serum. Increased concentrations of AST were found at 48 h but not at 24 h p.i. Early antibiotic therapy prevented increase of AST levels in infected mice. Late antibiotic treatment had no beneficial effect on AST concentrations (Fig. 4c, Additional file 2: Figure S3F). The urea concentration increased following early antibiotic therapy whereas no differences were detected following late intervention (Additional file 2: Figure S9C).

### Early antibiotic intervention protected alveolar–epithelial barrier integrity and reduced VEGF levels

**Fig. 5:**
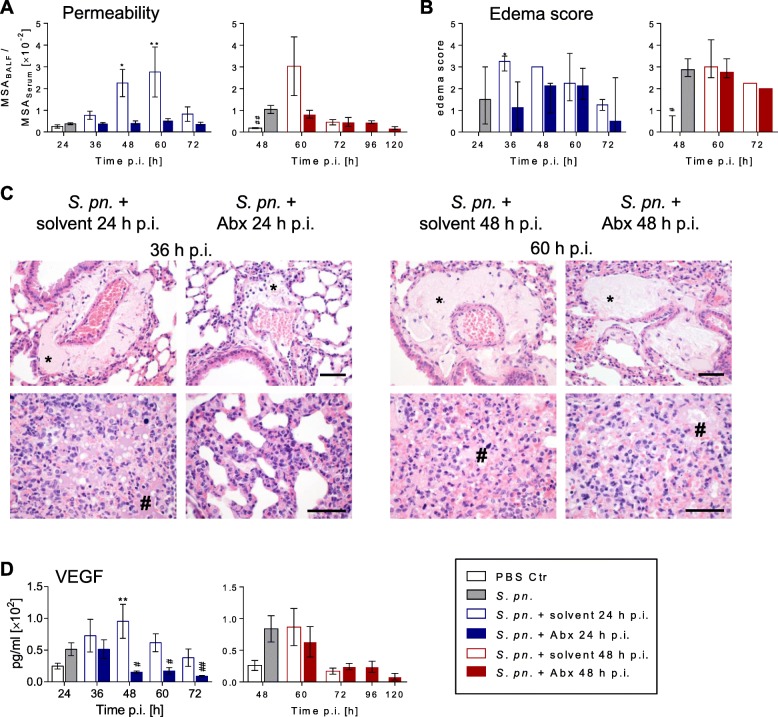
Early antibiotic treatment reduced lung barrier failure and VEGF levels. Mice were infected with *S. pneumoniae* and assigned equally to groups and analysis time points (**a**, **d** *n*_total_ = 9, **b**, **c** *n*_total_ = 4 per time point)*.* Starting 24 h or 48 h p.i., intervention groups were treated with ampicillin. As controls, mice were sham infected (PBS; **a**, **d** *n*_total_ = 7, **b**, **c** *n*_total_ = 4 per time point) or treated with solvent (0.9% NaCl). Mice surviving until designated analysis time point were sacrificed for BAL and blood sampling (number analyzed per time point presented in Additional file 1: Table S1) or histopathological analysis (number analyzed per time point presented in Additional file 1: Table S2). **a** Ratios of mouse serum albumin (MSA) BALF/serum reflecting lung barrier integrity, calculated from ELISA readouts. **b** Lung edema score, calculated from specified histopathological parameters displaying perivascular and alveolar edema formation. Results pooled from two independent experiments per time point. Median and 25–75% interquartile range. **c** Representative H&E staining. *Perivascular edema, #alveolar edema. Scale bars: 100 μm. **d** VEGF levels measured in BAL fluid by multiplex analysis. **a**, **d** Results pooled from three independent experiments per time point. Mean ± SEM. Two-way ANOVA/Sidak’s multiple comparisons test for comparison of ampicillin versus solvent treatment. **a**, **b** Kruskal–Wallis test/Dunn’s multiple comparisons test and **d** one-way ANOVA/Dunnett’s multiple comparisons test for comparison to *S. pneumoniae*-infected mice at therapy start. *Significant difference between groups at time point, #significant difference from therapy start: **a**, **b**, **d** *^/#^*p* < 0.05, **^/##^*p* < 0.01. Abx antibiotics, BALF bronchoalveolar lavage fluid, Ctr control, PBS phosphate buffered saline, p.i. post infection, *S*. *pn*. *Streptococcus pneumoniae*

Measurements of the alveolar–capillary barrier permeability index allow estimation of the degree of vascular leakage into alveolar spaces. Prior to treatment of infected mice, vascular leakage was evident at 48 h but not at 24 h p.i. Early antibiotic treatment successfully prevented development of vascular leakage observed in *S. pneumoniae*-infected solvent-treated control mice (Fig. 5a, Additional file 2: Figure S3G). In contrast, late antibiotic therapy failed to significantly reverse established lung permeability. In line with this finding, histopathological analysis of lung tissue integrity revealed significantly reduced interstitial and alveolar edema formation in early antibiotic-treated animals compared to solvent-treated controls. However, late antibiotic therapy did not reduce edema formation (Fig. 5b, c, Additional file 2: Figures S3H and S10A, B). Vascular endothelial growth factor (VEGF) and binding leukocytes trigger destabilization of endothelial cell–cell contacts [31]. VEGF levels in BALF of *S*. *pneumoniae*-infected mice were modestly elevated compared to PBS-infected control groups at the time points 24 h and 48 h p.i. Only after early ampicillin therapy did alveolar VEGF concentrations significantly decrease compared to the late treatment start and solvent-treated control groups (Fig. 5d, Additional file 2: Figure S3G).

## Discussion

Upon CAP diagnosis, commencement of immediate treatment is considered critical since a delay increases the likelihood of lethality [7, 9]. However, some patients present themselves in a phase of advanced disease or diagnosis is belated. In these cases, antibiotic treatment can become insufficient to avoid development of acute lung injury; thus, adjuvant therapeutic options are needed. Understanding the pathophysiology of acute lung injury in pneumonia with delayed treatment is a prerequisite to successful therapy development. Therefore, we established a mouse model of CAP comprising early and late antibiotic treatment of bacterial pneumonia mirroring different treatment commencements in CAP patients. Validity of the model was achieved as: severe pneumonia was established before early and late treatment start; treatment success rates in terms of survival outcome depended on timely treatment; and antibiotic treatment efficacy in terms of bacterial elimination was independent of timely treatment. Our model settings are thus in line with observations made in immunocompetent ICU patients with CAP who received an adequate antibiotic regimen and management of comorbidities, but nonetheless showed high mortality rates [32, 33].

Aiming to identify host-related factors responsible for detrimental disease outcome, we suspected an inadequate immune response, as described and reviewed by Mizgerd [34], as the underlying mechanism following late therapy. Indeed, histopathological analyses revealed that only late antibiotic treatment failed to reduce neutrophilic infiltration into the pleura and adjacent mediastinal adipose tissue. In contrast, recruitment of inflammatory cells into lung tissue and air spaces appeared unaffected by early and late antibiotic interventions but, nevertheless, alveolar levels of inflammatory mediators were found to be repressed.

In order to assist interpreting these confounding observations, we implemented our data in a cytokine–cell network model. The network analysis illustrated that antibiotic treatment suppressed proinflammatory cytokines more efficiently than inflammatory chemokines (such as CXCL1, CXCL5 and GM-CSF). This indicates that recruiting mechanisms like chemokine gradients were less affected by antibiotic treatment. However, pronounced antibiotic-dependent reduction in inflammatory mediators suggests that, contrary to cellular numbers, the activation state and effector functions of alveolar immune cells were dampened by antibiotic-governed bacterial eradication.

An intact blood–air barrier is crucial for gas exchange, and fatal barrier breakdown during streptococcal pneumonia, leading to acute respiratory distress syndrome (ARDS), is well described (reviewed in [35–37]). Accordingly, we observed that an intact lung barrier was associated with favorable therapy outcome in antibiotic-treated mice exhibiting severe pneumococcal pneumonia. Furthermore, differences in survival correlated with extended exposure time to pneumococci, resulting in a prolonged local and systemic contact with infection-induced inflammatory mediators. In line, early as opposed to late treatment prevented systemic inflammation.

The evident local and systemic inflammation might as well have occurred due to release of proinflammatory cytosolic components upon cell death processes. Necroptosis, a form of programmed necrosis, can be initiated by *S. pneumoniae* in pulmonary immune cells like macrophages and epithelial cells [38, 39]. In our pneumonia model, alveolar macrophage numbers remained stable upon infection, whereas numbers of inflammatory macrophages significantly increased with infection time, without showing significant differences between treatment and control groups. Due to their slow replenishment rates [40], stable numbers of alveolar macrophages rather speak against necroptosis. Monocyte-derived macrophages that were actively recruited to infected lung tissue could potentially have undergone necroptosis and been replaced by newly recruited cells. To date, it remains likely but unproven that higher rates of monocyte-derived macrophage and epithelial cell necroptosis, following a late antibiotic regimen, are one of the underlying mechanisms for elevated inflammation and vascular leakage. Correspondingly, the presence of PAMPs as well as necroptosis-independent DAMPS could have likely regulated the activation status of local and systemic immune cells, thereby altering levels of inflammatory mediators following antibiotic intervention.

In severe pneumonia, only early antibiotic therapy, administered prior to barrier breakdown, prevented systemic inflammation, development of pleuritis, steatitis and elevated AST levels, which was followed up by restoration of fitness and rescue of mice from fatal outcome. Gracia et al. [41] have shown in *S. pneumoniae*-infected rats that early and late antibiotic regimens similarly eliminated bacterial burdens whereas only early therapy helped to prevent lung damage. However, the authors started early therapy just 1 h p.i., an important difference to our protocol whereby infected mice already developed pneumonia prior to antibiotic treatment. We therefore conclude that our model represents the patients’ situation in a more compatible fashion.

Nonetheless, our mouse model remains a species-different approach to a highly complex human disease. It does not include therapeutic measures conducted in the ICU such as additional intake of fluids, (high flow) oxygen or macrolide therapy. Furthermore, laboratory mice which did not experience pathogen contact before generally show a modified immune response as humans do, for example, since they are missing mucosal memory cells [42]. Nevertheless, mice exhibited many parallels to the patients’ course of infection; from a contained local infection, pneumonia developed into life-threatening systemic inflammation as often seen in CAP patients. Further studies from our laboratory likewise described processes in murine pneumonia which resembled the human course of pneumonia [43–45].

As a consequence, we stress the importance of defining CAP as an emergency and to identify and treat CAP patients at risk prior to lung barrier failure and systemic inflammation [13, 14, 46]. Besides many promising studies [47–52], specific adjunctive therapies for CAP are still missing. Such therapies should be coadministered with antibiotics in order to reduce lung barrier failure, thereby alleviating the disease course and finally reducing mortality and the length of hospital stay.

Our mouse model of antibiotic-treated CAP will aid in investigating how barrier-stabilizing interventions can prevent fatal disease progression in mice. Conclusively, proof-of-concept analyses indicating to which extent this mouse model adequately mirrors a significant range of patients must follow.

## Conclusions

By combining a mouse model of severe pneumococcal pneumonia with antibiotic treatment, we identified host factors of high relevance for successful therapy outcome. We were able to show that antibiotic treatment effectively reduces local pulmonary inflammation independent of treatment start and without beneficial effect on survival rates. However, despite being unable to reverse lung barrier dysfunction, lung edema and systemic inflammation once established, timely antibiotic intervention effectively prevented their development, significantly increasing survival. Handling CAP as an emergency along with new lung barrier stabilizing therapies might thus aid in finally lowering mortality rates.

## Additional files

Additional file 1: Supplementary Materials and methods. **Table S1.** Total numbers of mice analyzed per group per analysis time point. **Table S2.** Total numbers of mice analyzed per group per histopathological analysis time point. **Table S3.** Murine pneumonia scoring system. **Figure S1.** Infection, antibiotic regimen and analysis time points (PDF 272 kb)

Additional file 2: **Figure S2.** Body temperature and histopathological analysis. **Figure S3.** Early versus late antibiotic regimen at 60 h and 72 h p.i. **Figure S4.** Innate immune cell gating strategy. **Figure S5.** Innate immune cell analysis in BAL and blood. **Figure S6.** Chemokine and cytokine levels in BAL fluid. **Figure S7.** Histopathological analysis of lung inflammation. **Figure S8.** Cytokine–cell network. **Figure S9.** Cytokine, chemokine and urea levels in serum. **Figure S10.** Histopathological analysis of edema development (PDF 1638 kb)

## Abbreviations

ARDS: Acute respiratory distress syndrome
AST: Aspartate aminotransferase
BAL: Bronchoalveolar lavage
BALF: Bronchoalveolar lavage fluid
CAP: Community-acquired pneumonia
CFU: Colony forming unit
ELISA: Enzyme-linked immunosorbent assay
G-CSF: Granulocyte colony-stimulating factor
GM-CSF: Granulocyte–macrophage colony-stimulating factor
H&E: Hematoxylin and eosin
ICU: Intensive care unit
IFN: Interferon
IL: Interleukin
MSA: Mouse serum albumin
p.i.: Post infection
PAMP: Pathogen-associated molecular pattern
PBS: Phosphate buffered saline
PMN: Polymorphonuclear leukocyte
TNF: Tumor necrosis factor
VEGF: Vascular endothelial growth factor
