# Supplementary material for: Delay in antibiotic therapy results in fatal disease outcome in murine pneumococcal pneumonia
Source: Crit Care. 2018 Nov 1;22:287. doi: 10.1186/s13054-018-2224-5 (PMC6211394; doi:10.1186/s13054-018-2224-5)
Supplement: Supplementary file 1 — Supplementary Materials and methods. Table S1. Total numbers of mice analyzed per group per analysis time point. Table S2. Total numbers of mice analyzed per group per histopathological analysis time point. Table S3. Murine pneumonia scoring system. Figure S1. Infection, antibiotic regimen and analysis time points (PDF 272 kb) [file 13054_2018_2224_MOESM1_ESM.pdf]

Delay in Antibiotic Therapy Results in Fatal Disease Outcome in Murine Pneumococcal Pneumonia

Sarah Berger, Cengiz Gökeri, Shishir K. Gupta, Julio Vera, Kristina Dietert, Ulrike Behrendt, Jasmin Lienau, Sandra-Maria Wienhold, Achim D. Gruber, Norbert Suttorp, Martin Witzenrath*; Geraldine Nouailles*

* M.W. and G.N. contributed equally to this work

ONLINE DATA SUPPLEMENT – File 1/2

Materials and Methods

Study approval. Female C57BL/6N mice (8 to 10 weeks, 18 to 20 g; Charles River, Sulzfeld, Germany), housed under specific-pathogen-free conditions with a 12 h light dark rhythm and food and water ad libitum, were used in all experiments. All animal procedures were approved by local institutional (Charité – Universitätsmedizin Berlin) and governmental (Landesamt für Gesundheit und Soziales (LaGeSo) Berlin) authorities. Animal housing and experimental procedures complied with the Federation of European Laboratory Animal Science Associations (FELASA) guidelines and recommendations for the care and use of laboratory animals, and all efforts were made to minimize animal discomfort and suffering.

Bacteria and infection of mice. Bacteria (*S. pn.* serotype 3, NCTC7978) were freshly prepared at the day of infection. Liquid THY media + 0.5 % yeast was used to cultivate *S. pn.* at 37 °C until the bacteria reached logarithmic growth phase, harvested and resuspended in PBS. Mice were anesthetized by intraperitoneal injection of ketamine (80 µg/g body weight) and xylazine (25 µg/g body weight) and transnasally inoculated as described in detail [1] with 5×10^6 colony forming units (CFU) *S. pn* in 20 µl PBS. Control mice received sham infection (20 µl PBS). Body weight and temperature were measured at 12 h intervals.

Antibiotic therapy. Starting at 24 h or 48 h p.i., ampicillin (0.4 mg/mouse) or 0.9 % NaCl (solvent-control) was injected intraperitoneally every 12 h. A flow diagram illustrating time course of infection, therapy and analysis time points can be taken from Figure S1.

Experimental procedure. Mice that met the inclusion criteria at 24 h p.i. (body weight loss more than 10 % and/or body temperature < 37.0°C) and reached the designated endpoints were anesthetized intraperitoneally with ketamine (80 mg/kg) and xylazine (25 mg/kg), exsanguinated by puncture of the vena cava and analyzed as described in the next section. The number of mice analyzed per group per analysis time point are described in Table S1 and S2. Number of excluded animals: n = 3.

Clinical scoring. Since mice do not develop typical human-like pneumonia symptoms like coughing or sputum production, we assessed specific murine pneumonia symptoms. Murine pneumonia symptoms were defined by experienced veterinarians and assessed in all experimental groups at 12 h intervals by rating the appearance and behavior of the mice on a scale of 0 (absent) to 1 (present) and 2 (severe), including: appearance of the fur and eyes, abnormalities in behavior, breathing rate, and symptoms of dehydration. Summary of scored symptoms provides an individual clinical score per mouse. The details of the scoring system are depicted in Table S3.

Blood and serum. 50 µl of blood were stored in EDTA Tubes (Sarstedt) at 4 °C for hemogram (measured with Scil Vet abc, scil animal care company GmbH) and bacterial load. Remaining blood was collected for serum extraction in Serum Separator Tubes (BD) and left for 30 minutes at room temperature, followed by centrifugation at 12,000 × g for 3 minutes. Sera were stored at -80 °C until analysis.

Histological analysis. Mice (Table S2) were sacrificed, trachea was ligated prior to opening of the thorax to prevent alveolar collapse. Lungs were carefully removed and immediately immersion-fixed in 4 % buffered formalin, embedded in paraffin, cut into 2 µm sections, and stained with hematoxylin and eosin (H & E) as described [2, 3]. Per lung, three evenly distributed sections were microscopically evaluated by a board-certified veterinary pathologist to assess dissemination and quality of pathologic alterations using specified lung inflammation parameters (total lung area affected; distribution of lung lesions; peribronchial, interstitial, and intra-alveolar inflammation; alveolar necrosis, perivascular inflammation and edema, infiltration by neutrophils and macrophages; alveolar edema and hemorrhage, pleuritis, and steatitis) [4, 5]. The lung inflammation score was expressed as the sum of the following relevant parameters graded on a scale of 0 (absent) to 4 (severe): degree of inflammation, infiltration of neutrophils, pleuritis, and steatitis. The lung edema score was expressed as the sum of distribution and degree of interstitial (perivascular) and alveolar edema graded on a scale of 0 (absent) to 5 (massive).

Leukocyte differentiation in BAL. Cells were pre-incubated with blocking antibody (Anti-CD16/32, BD). The following antibodies were used for the cell surface staining: CD11c-Cy5 (N418, ATCC), CD11b-PE-Cy7 (M1/70, eBioscience), F4/80-PE (BM8, eBioscience), Ly6G-PerCP-Cy5.5 (1A8, BD), Ly6C-V450 (AL-21, BD) and MHCII-Alexa Fluor 700 (M5/114.15.2, eBioscience). Stained samples were measured using the BD FACS Canto II and analyzed with BD FACSDiva software. Cell numbers were calculated using CountBright Absolute Counting Beads (Thermo Fisher Scientific).

Cytokine multiplex assays and ELISA. Mouse cytokine/chemokine levels (TNF-α, IFN-γ, IL-6, IL-1β, G-CSF, GM-CSF, CCL2, CCL3, CXCL1 and CXCL5) were measured in BALF and serum with the ProcartaPlex Custom Mix & Match according to the manufacturer's

instructions (Affymetrix Bioscience). Serum samples were analyzed in a 1:2 dilution, BALF samples undiluted. BALF levels of CXCL2 and CXCL5 were measured by ELISA (RD), according to the manufacturer's instructions.

Cytokine-immune cell network. A three-layered network was constructed focusing on the connections between measured immune cells and chemokines/cytokines. The diagram editor CellDesigner was used for visualizing bacterial loads plus connections between cells and mediators, connected by their receptors to the time of therapy start (24 h / 48 h) and in the time course of antibiotic treatment or solvent control. To facilitate data visualization, the network was translated into the bioinformatics software platform Cytoscape [6]. Protein components of the network were annotated using Uniprot identifiers. For the connections between immune cells with their mediators, Pubmed identifiers of papers indicating the interaction were used.

Table S1. Total numbers of mice analyzed per group per analysis time point

Table depicts total numbers of mice analyzed at individual time points and corresponding groups. The table applies to Figures 1, 2, 3, 4, 5, and Figures S2, S3, S5, S6, S9.

Time p.i. (h)	PBS	*S. pn.* ST3	*S. pn.* ST3 + ampicillin 24 h p.i.	*S. pn.* ST3 + solvent 24 h p.i.	*S. pn.* ST3 + ampicillin 48 h p.i.	*S. pn.* ST3 + solvent 48 h p.i.
24	7	9				
36	7		9	7		
48	7	9	9	7		
60	7		9	7	7	7
72	7		9	2	5	2
96			9		3	
120			7		2	

Table S2. Total numbers of mice analyzed per group per histo-pathological analysis time point. Table depicts total numbers of mice analyzed at individual time points and corresponding groups. The table applies to Figures 3 and 5, and Figures S2, S3, S7, and S10.

Time p.i. (h)	PBS	*S. pn.* ST3	*S. pn.* ST3 + ampicillin 24 h p.i.	*S. pn.* ST3 + solvent 24 h p.i.	*S. pn.* ST3 + ampicillin 48 h p.i.	*S. pn.* ST3 + solvent 48 h p.i.
**24**	**4**	**4**				
**36**			**4**	**4**		
**48**	**4**	**4**	**4**	**3**		
**60**			**4**	**4**	**4**	**3**
**72**			**4**	**2**	**2**	**2**

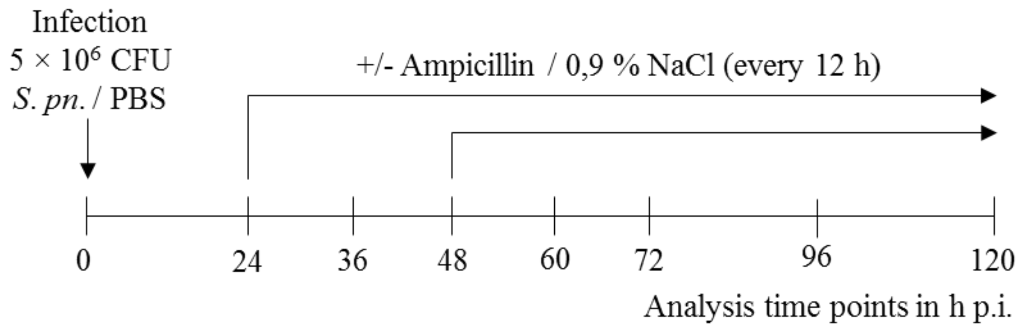

Figure S1: Infection, antibiotic regimen and analysis time points.

Table S3. Murine pneumonia scoring system

**Symptom**	**Score**
**Fur**	
Shiny	0
Disheveled	1
**Eyes**	
Bright	0
Purulent, weeping	1
**Behavior**	
Curious, awaken	0
Calm/Self-isolation	1
Apathy	2
**Breathing**	
Steady, consistent	0
Hyper- /hypoventilation	1
Laboured	2
**Pain**	
No indication	0
Seizures, lurching, hunched posture	1
**Dehydration**	
No indication	0
Lingering cutaneous folds	1

Supplemental References

1. Saleh M, Abdullah MR, Schulz C, Kohler T, Pribyl T, Jensch I, Hammerschmidt S. Following in real time the impact of pneumococcal virulence factors in an acute mouse pneumonia model using bioluminescent bacteria. J Vis Exp 2014;e51174.
2. Reppe K, Radunzel P, Dietert K, Tschernig T, Wolff T, Hammerschmidt S, Gruber AD, Suttorp N, Witzenrath M. Pulmonary immunostimulation with MALP-2 in influenza virus-infected mice increases survival after pneumococcal superinfection. Infect Immun 2015;83:4617-4629.
3. Dames C, Akyuz L, Reppe K, Tabeling C, Dietert K, Kershaw O, Gruber AD, Meisel C, Meisel A, Witzenrath M, Engel, O. Miniaturized bronchoscopy enables unilateral investigation, application, and sampling in mice. Am J Respir Cell Mol Biol 2014;51:730-737.
4. Dietert K, Gutbier B, Wienhold SM, Reppe K, Jiang X, Yao L, Chaput C, Naujoks J, Brack M, Kupke A, et al: Spectrum of pathogen- and model-specific histopathologies in mouse models of acute pneumonia. PLoS One 2017;12:e0188251.
5. Dietert K, Reppe K, Mundhenk L, Witzenrath M, Gruber AD: mCLCA3 modulates IL-17 and CXCL-1 induction and leukocyte recruitment in murine Staphylococcus aureus pneumonia. PLoS One 2014;9:e102606.
6. Shannon P, Markiel A, Ozier O, Baliga NS, Wang JT, Ramage D, Amin N, Schwikowski B, Ideker T. Cytoscape: a software environment for integrated models of biomolecular interaction networks. Genome Res 2003;13:2498-2504.
