# Supplementary material for: Delay in antibiotic therapy results in fatal disease outcome in murine pneumococcal pneumonia
Source: Crit Care. 2018 Nov 1;22:287. doi: 10.1186/s13054-018-2224-5 (PMC6211394; doi:10.1186/s13054-018-2224-5)
Supplement: Supplementary file 2 — Figure S2. Body temperature and histopathological analysis. Figure S3. Early versus late antibiotic regimen at 60 h and 72 h p.i. Figure S4. Innate immune cell gating strategy. Figure S5. Innate immune cell analysis in BAL and blood. Figure S6. Chemokine and cytokine levels in BAL fluid. Figure S7. Histopathological analysis of lung inflammation. Figure S8. Cytokine–cell network. Figure S9. Cytokine, chemokine and urea levels in serum. Figure S10. Histopathological analysis of edema development (PDF 1638 kb) [file 13054_2018_2224_MOESM2_ESM.pdf]

Delay in Antibiotic Therapy Results in Fatal Disease Outcome in Murine Pneumococcal Pneumonia

Sarah Berger, Cengiz Gökeri, Shishir K. Gupta, Julio Vera, Kristina Dietert, Ulrike Behrendt, Jasmin Lienau, Sandra-Maria Wienhold, Achim D. Gruber, Norbert Suttorp, Martin Witzenrath*; Geraldine Nouailles*

* M.W. and G.N. contributed equally to this work

ONLINE DATA SUPPLEMENT – File2/2

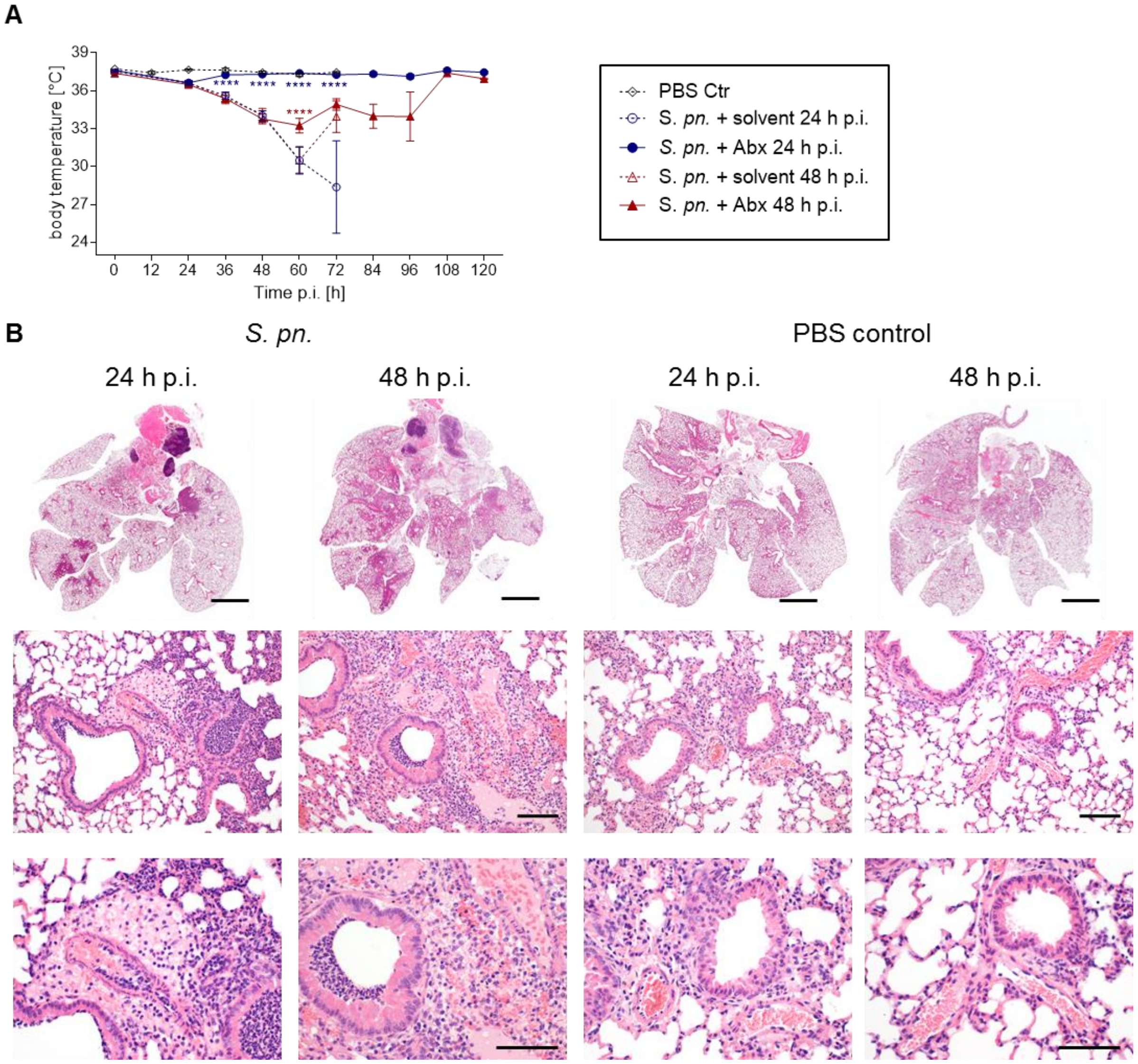

Figure S2. Body temperature and histopathological analysis. Mice were infected with *S. pn.* and assigned equally to groups and analysis time points (A: $n_{total} = 9$; B: $n_{total} = 4$ per time point). Starting 24 h or 48 h p.i., intervention groups were treated with ampicillin. As controls, mice were sham-infected (PBS, A: $n_{total} = 7$; B: $n_{total} = 4$ per time point) or treated with solvent (0.9 % NaCl). **A,** Body temperature was assessed for all mice until designated analysis time point. **B,** Mice surviving until designated analysis time point were sacrificed for histo-pathological analysis (n analysed per time point listed in Additional File 1, Table S2). **A**, Body temperature curves. Means ± SEM, 2-way ANOVA/Sidak's multiple comparisons test for comparison of ampicillin- versus solvent-treatment. **** $p < 0.0001$. **B**, 2 µm lung sections were stained with hematoxylin and eosin for histopathological analysis. Images representative of two independent experiments. Scale bars: whole lungs (top): 2 mm; magnified slices (bottom): 100 µm.

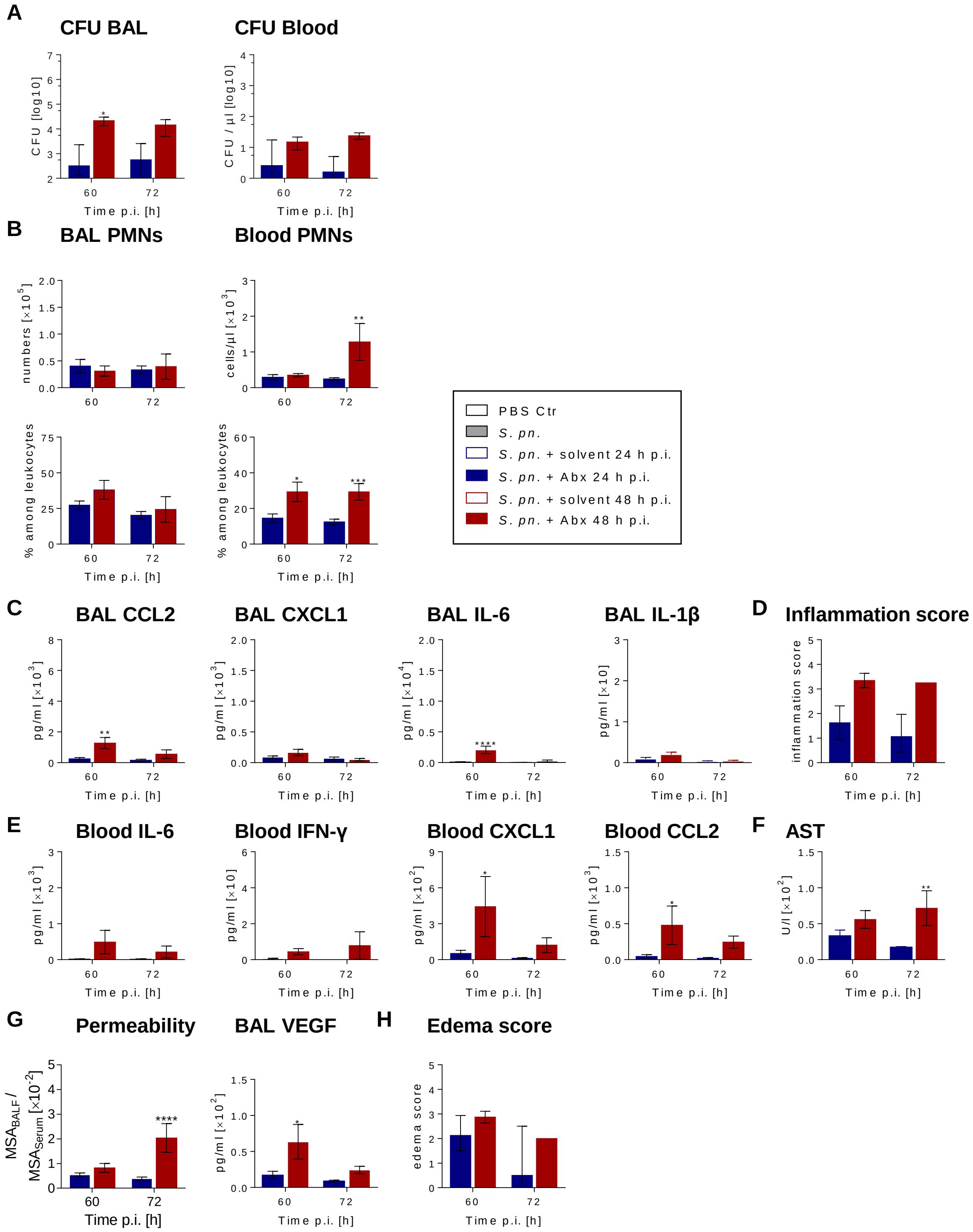

Figure S3. Direct comparison of between early versus late antibiotic regimen at 60 h and 72 h p.i.. Mice were infected with *S. pn.* and assigned equally to groups and analysis time points (A - C, E - G: n_{total} = 9; D, H: n_{total} = 4 per time point). Starting 24 h or 48 h p.i., intervention groups were treated with ampicillin. As controls, mice were sham-infected (PBS, (A - C, E - G: n_{total} = 7; D, H: n_{total} = 4 per time point)) or treated with solvent (0.9 % NaCl). Mice surviving

until designated analysis time point were sacrificed for BAL and blood sampling (A - C, E - G: n analyzed per time point listed in Additional File 1, Table S1) or histo-pathological analysis (D, H: n analyzed per time point listed in Additional File 1, Table S2). Results were pooled from two (D, H) or three (A - C, E - G) independent experiments per time point. **A,** Bacterial burden in BAL and blood of respective mice analyzed at indicated time points. **B**, Numbers and frequencies of PMNs in BAL of respective mice analyzed at indicated time points p.i., as quantified by flow cytometry and numbers and frequencies of PMNs measured in EDTA-blood by Scil Vet abc hematology analyser of respective mice. **C,** Chemokine and cytokine protein levels in BAL fluid measured by multiplex analysis or ELISA. **D,** Lung inflammation score, calculated from specified histopathological parameters displaying distribution, severity, and main character of lung lesions. **E,** Cytokine and chemokine protein levels in serum measured by multiplex analysis. **F,** Serum AST levels measured by Cobas 8000 C701. **G**, Ratios of mouse serum albumin (MSA) (BALF) / MSA (serum) reflecting lung barrier integrity, calculated from ELISA readouts and BAL VEGF levels measured in BAL fluid by multiplex analysis. **H**, Lung edema score, calculated from specified histopathological parameters displaying perivascular and alveolar edema formation. **A, D, H:** Medians and 25-75 % IQR, **B, C, E - G:** Means ± SEM. **A - H**, 2-way ANOVA/Sidak's multiple comparisons test for comparison of ampicillin- versus solvent-treatment. $^{*}p< 0.05$, $^{**}p< 0.01$ $^{***}p< 0.001$ and $^{****}p< 0.001$.

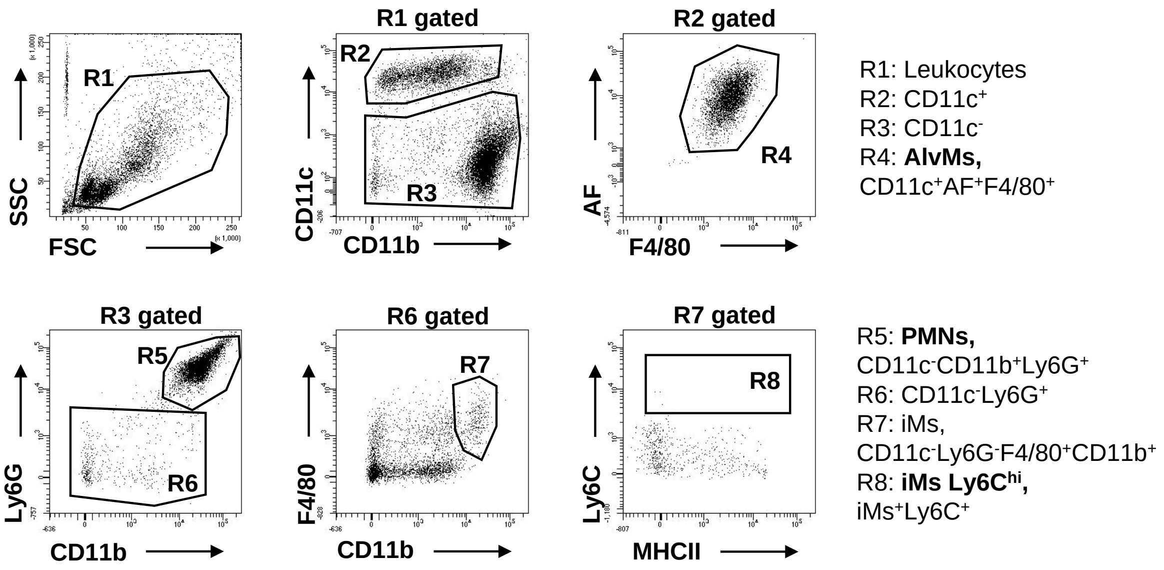

Figure S4. Innate immune cell gating strategy. Dot blots indicating flow cytometric gating strategy of innate immune cell populations in alveolar spaces.

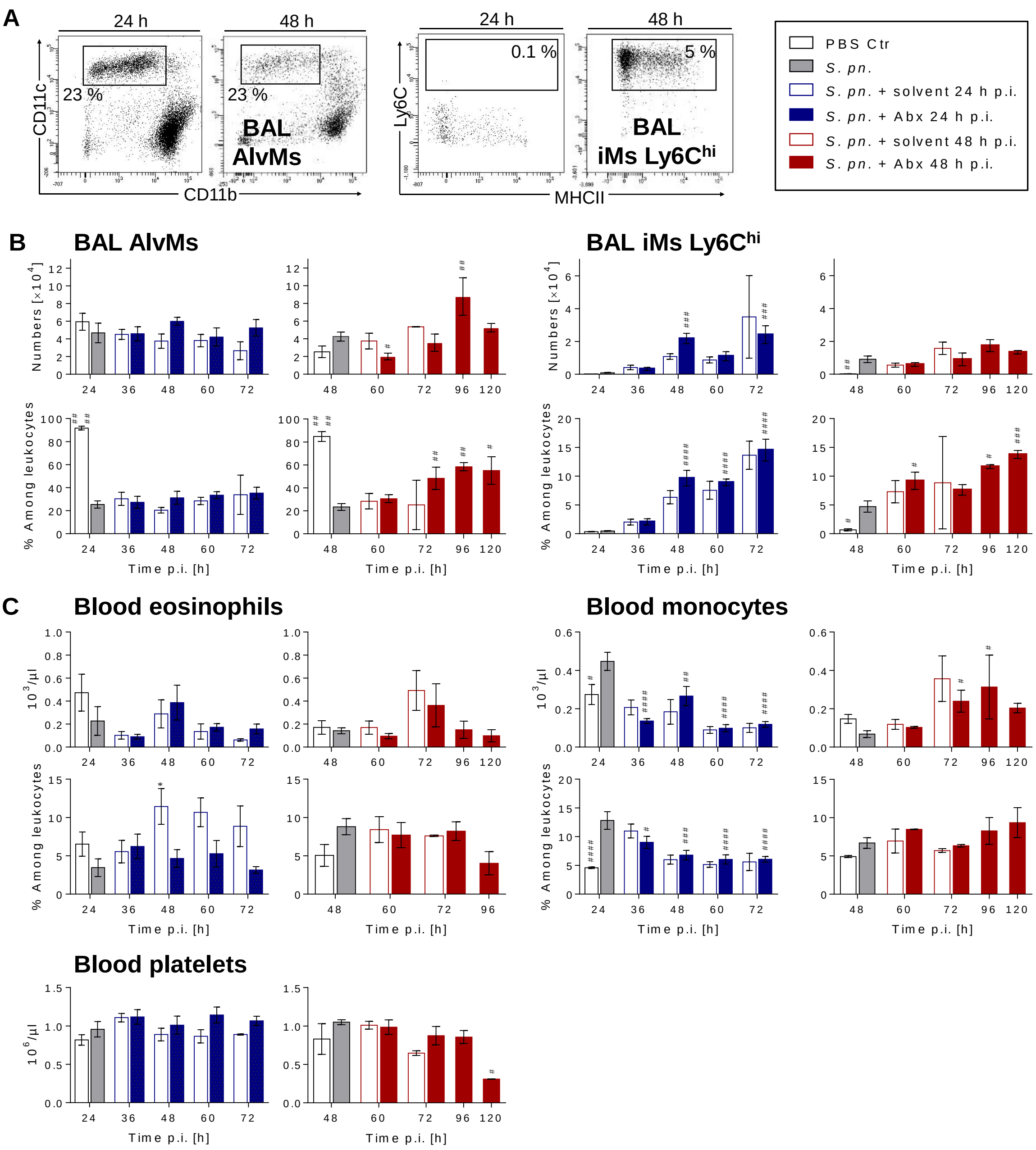

Figure S5. Innate immune cell analysis in BAL and blood. Mice were infected with *S. pn.* and assigned equally to groups and analysis time points (n_{total} = 9 per time point). Starting 24 h or 48 h p.i., intervention groups were treated with ampicillin. As controls, mice were sham-infected (PBS, n_{total} = 7 per time point) or treated with solvent (0.9 % NaCl). Mice surviving until designated analysis time point were sacrificed for BAL and blood sampling (n analyzed per time point listed in Additional File 1, Table S1). **A**, Representative dot blots illustrating gating of innate BAL cell populations (alveolar macrophages (alvMs) and inflammatory macrophages (iMs Ly6C^{hi})) at 24 h and 48 h p.i. **B**, Numbers and frequencies of alvMs and iMs Ly6C^{hi} in BAL of respective mice surviving at indicated time points p.i.; determined by flow cytometry. **C**, Numbers and frequencies of eosinophils and monocytes and numbers of platelets

measured in EDTA-blood by Scil Vet abc hematology analyzer. **B, C,** Results were pooled from three independent experiments per time point. Means ± SEM, 2-way ANOVA/Sidak's multiple comparisons test for comparison of ampicillin- versus solvent-treatment. One-way ANOVA/Dunnett's multiple comparisons test for comparison to *S .pn.*-infected mice at therapy start. * indicates significant difference between groups at time point, # indicates significant difference to therapy start. $^{*/\#}$ $p < 0.05$, $^{\#\#}$ $p < 0.01$, $^{\#\#\#}$ $p < 0.001$ and $^{\#\#\#\#}$ $p < 0.0001$.

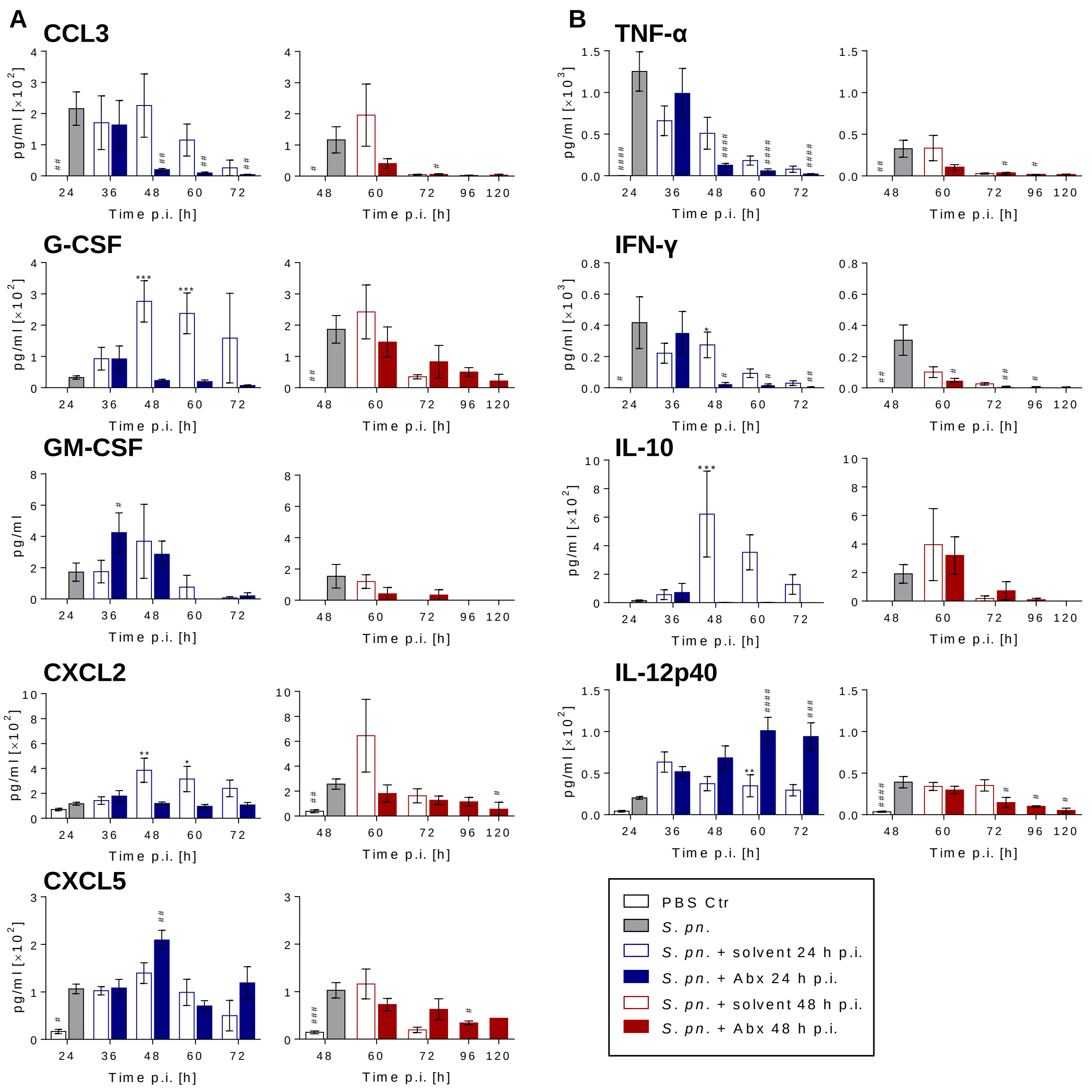

Figure S6. Chemokine and cytokine levels in BAL fluid. Mice were infected with *S. pn.* and assigned equally to groups and analysis time points (n_{total} = 9 per time point). Starting 24 h or 48 h p.i., intervention groups were treated with ampicillin. As controls, mice were sham-infected (PBS, n_{total} = 7 per time point) or treated with solvent (0.9 % NaCl). Mice surviving until designated analysis time point were sacrificed for BAL sampling (n analyzed per time point listed in Additional File 1, Table S1). **A**, Chemokine and **B**, cytokine protein levels in BAL fluid measured by multiplex analysis or ELISA. Results were pooled from three independent experiments per time point. Means ± SEM, 2-way ANOVA/Sidak's multiple comparisons test for comparison of ampicillin- versus solvent-treatment. One-way ANOVA/Dunnett's multiple comparisons test for comparison to *S. pn.*-infected mice at therapy start. * indicates significant difference between groups at time point, # indicates significant difference to therapy start. $^{*/\#}$ $p < 0.05$, $^{**/\#\#}$ $p < 0.01$, $^{***/\#\#\#}$ $p < 0.001$ and $^{\#\#\#\#}$ $p < 0.0001$.

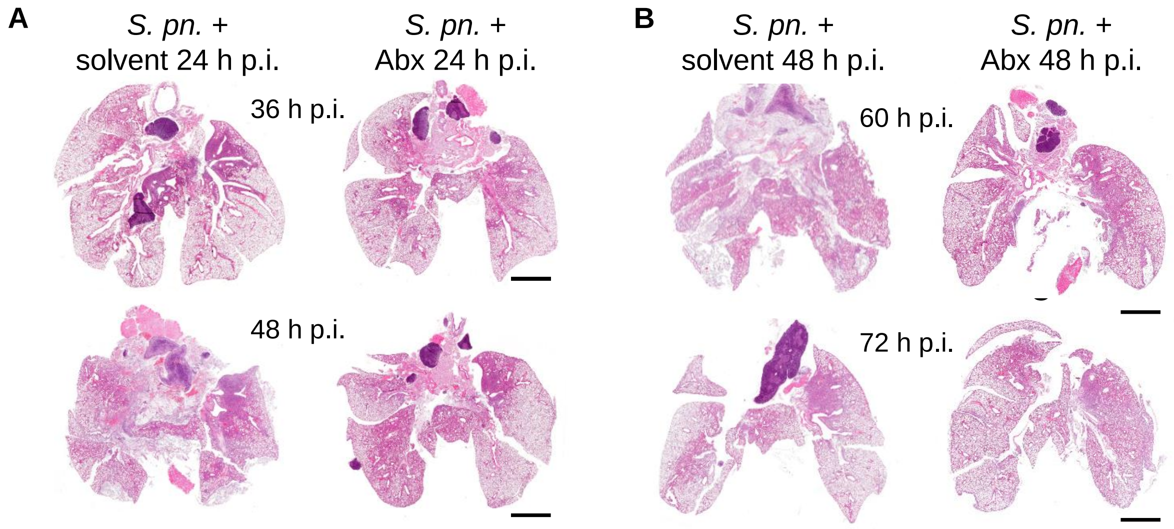

Figure S7. Histo-pathological analysis of lung inflammation. Mice were infected with *S. pn.* and assigned equally to groups and analysis time points (n_{total} = 4 per time point). Starting 24 h or 48 h p.i., intervention groups were treated with ampicillin. As controls, mice were sham-infected (PBS, n_{total} = 4 per time point) or treated with solvent (0.9 % NaCl). Mice surviving until designated analysis time point were sacrificed for histo-pathological analysis (n analyzed per time point listed in Additional File 1, Table S2). H & E stained whole lung sections for histological analysis. Images representative of two independent experiments. **A**, Early therapy start (24 h p.i.). **B**, Late therapy start (48 h p.i.). **A**, **B**, left: solvent-treatment (control), right: antibiotic treatment. Scale bars: 2 mm.

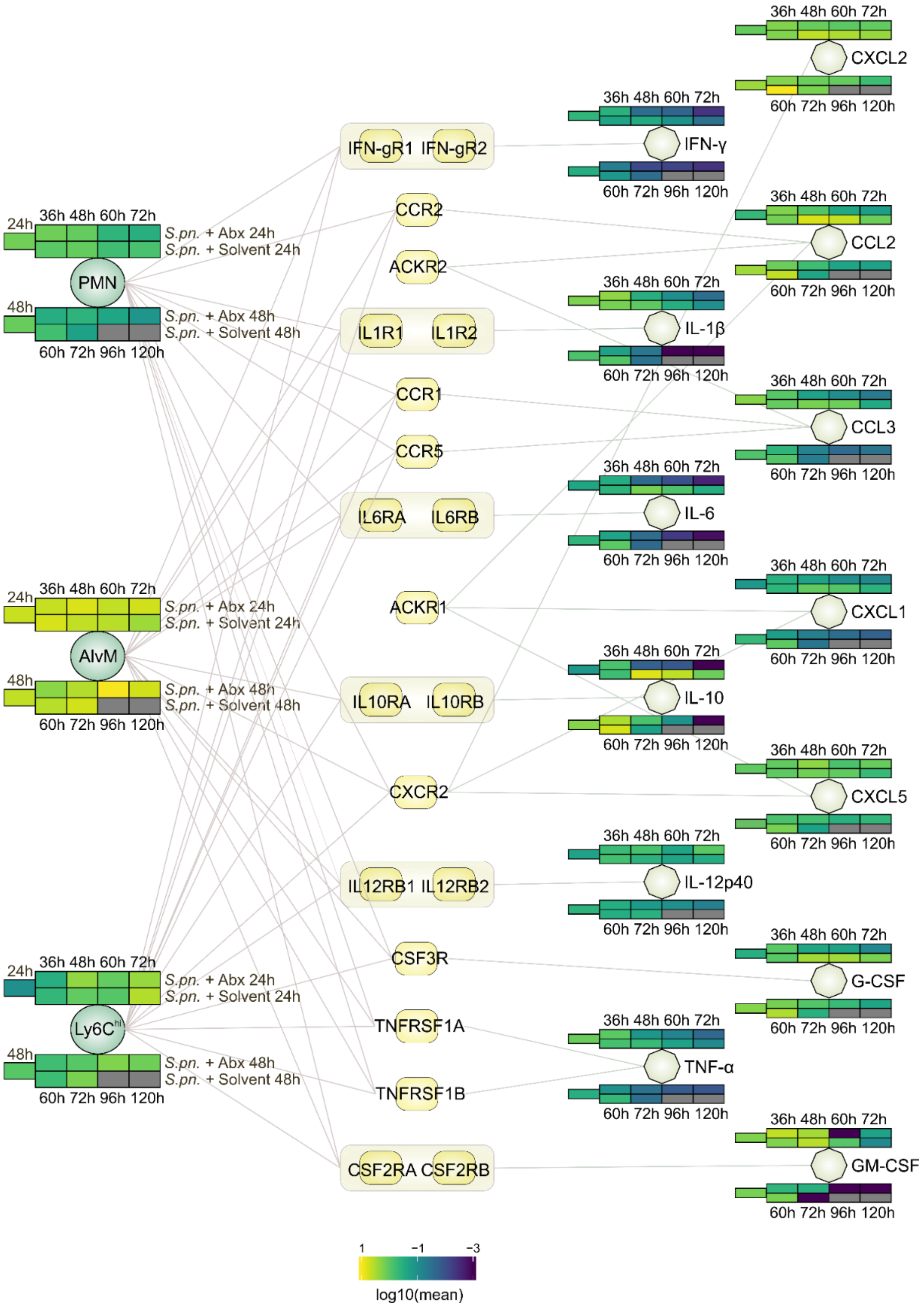

Figure S8. Cytokine-cell network. Visualization of analysis of alveolar immune cells (PMN, alvM, and Ly6C^{hi} iMs) and their receptor interactions with putative ligand cytokines and chemokines present in BAL fluid. Lines connect cells with their expressed receptors and receptors with their ligands. Grids illustrate cell numbers and cytokine/chemokine quantities in BAL prior therapy commencement (24 h/48 h) and at different time points following early (*S. pn.* + Abx 24 h) and late (*S. pn.* + Abx 48 h) antibiotic treatment and their respective control groups (*S. pn.* + solvent 24 h and *S. pn.* + solvent 48 h).

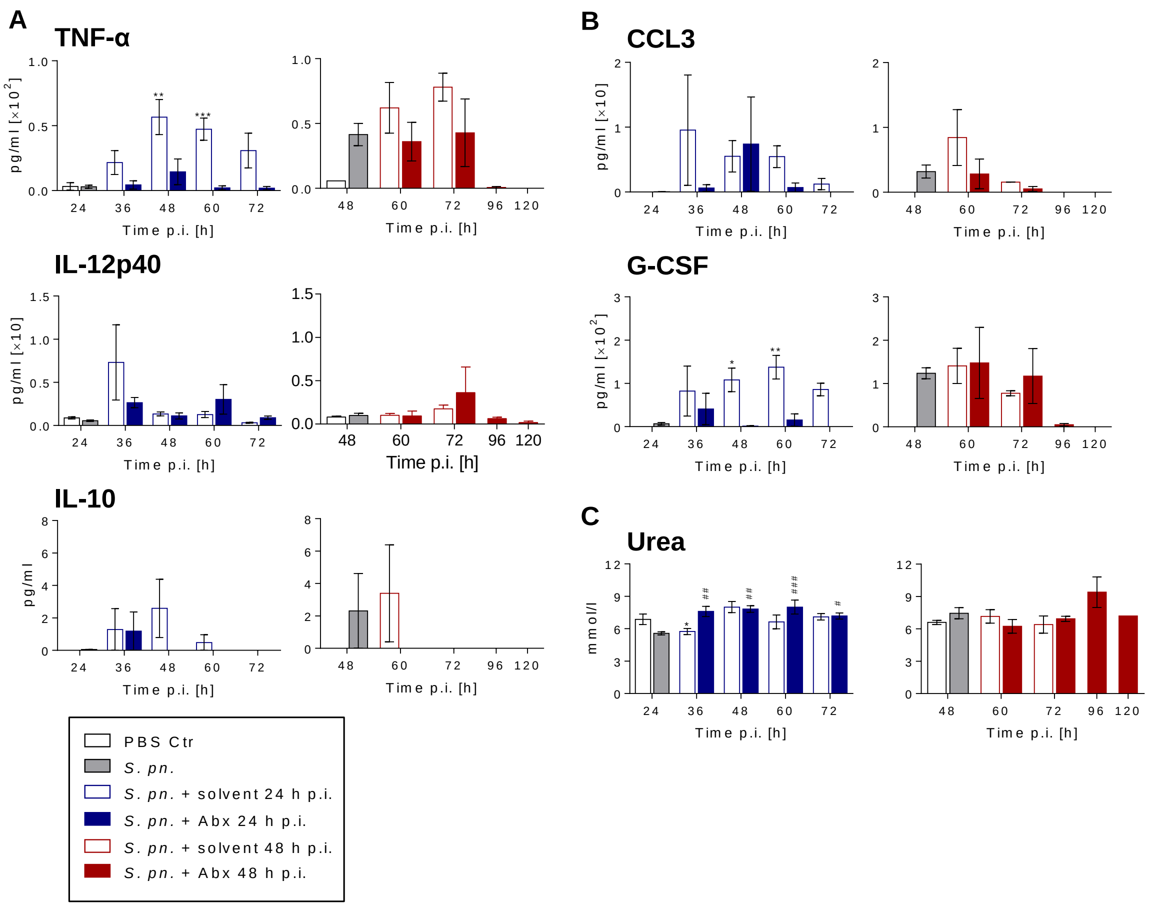

Figure S9. Cytokine, chemokine and urea levels in serum. Mice were infected with *S. pn.* and assigned equally to groups and analysis time points (n_{total} = 9 per time point). Starting 24 h or 48 h p.i., intervention groups were treated with ampicillin. As controls, mice were sham-infected (PBS, n_{total} = 7 per time point) or treated with solvent (0.9 % NaCl). Mice surviving until designated analysis time point were sacrificed for blood sampling (n analyzed per time point listed in Additional File 1, Table S1). **A**, Cytokine and **B**, chemokine protein levels in serum measured by multiplex analysis. **C**, Serum Urea levels measured by Cobas 8000 C701. **A - C,** Results were pooled from three independent experiments per time point. Means ± SEM, 2-way ANOVA/Sidak's multiple comparisons test for comparison of ampicillin- versus solvent-treatment. One-way ANOVA/Dunnett's multiple comparisons test for comparison to *S. pn.*-infected mice at therapy start. * indicates significant difference between groups at time point, # indicates significant difference to therapy start. $^{*/\#}$ $p < 0.05$, $^{**/\#\#}$ $p < 0.01$ and $^{***/\#\#\#}$ $p < 0.001$.

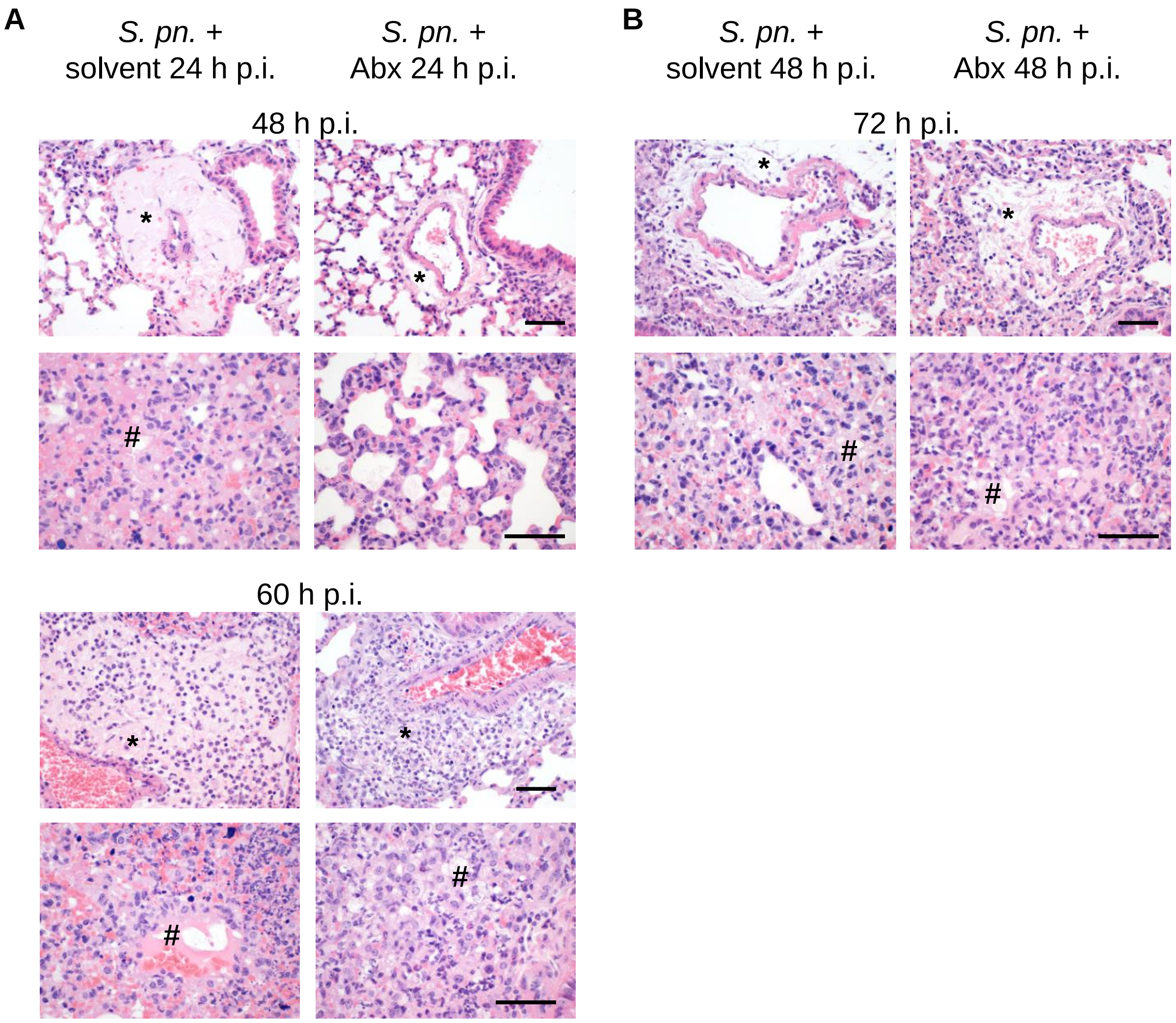

Figure S10. Histopathological analysis of edema development. Mice were infected with *S. pn.* and assigned equally to groups and analysis time points (n_{total} = 4 per time point). Starting 24 h or 48 h p.i., intervention groups were treated with ampicillin. As controls, mice were sham-infected (PBS, n_{total} = 4 per time point) or treated with solvent (0.9 % NaCl). Mice surviving until designated analysis time point were sacrificed for histo-pathological analysis (n analyzed per time point listed in Additional File 1, Table S2). H & E stainings representative of two independent experiments. **A**, Early therapy start (24 h p.i.). **B**, Late therapy start (48 h p.i.). **A**, **B**, left: solvent-treatment (control), right: antibiotic treatment. * indicates perivascular edema. # indicates alveolar edema. Scale bars: 100 µm.
